# Acupuncture Antiarrhythmic Effects on Drug Refractory Persistent Atrial Fibrillation: Study Protocol for a Randomized, Controlled Trial

**DOI:** 10.1155/2015/613970

**Published:** 2015-02-17

**Authors:** Jimin Park, Hyun Soo Kim, Seung Min Lee, Kanghyun Yoon, Woo-shik Kim, Jong Shin Woo, Sanghoon Lee, Jin-Bae Kim, Weon Kim

**Affiliations:** ^1^Department of Acupuncture and Moxibustion, College of Korean Medicine, Kyung Hee University, Seoul 130-701, Republic of Korea; ^2^Division of Cardiology, Department of Internal Medicine, College of Medicine, Kyung Hee University, Seoul 130-701, Republic of Korea

## Abstract

*Background.* Atrial fibrillation (AF) is the most common form of arrhythmia. Several trials have suggested that acupuncture may prevent AF. However, the efficacy of acupuncture for AF prevention has not been well investigated. Therefore, we designed a prospective, two-parallel-armed, participant and assessor blinded, randomized, sham-controlled clinical trial to investigate acupuncture in persistent AF (ACU-AF). *Methods.* A total of 80 participants will be randomly assigned to active acupuncture or sham acupuncture groups in a 1 : 1 ratio. Both groups will take the same antiarrhythmic medication during the study period. Patients will receive 10 sessions of acupuncture treatment once a week for 10 weeks. The primary endpoint is AF recurrence rate. Secondary endpoints are left atrium (LA) and left atrial appendage (LAA) changes in function and volume, and inflammatory biomarker changes. *Ethics.* This study protocol was approved by the institutional review boards (IRBs) of Kyung Hee University Hospital (number 1335-04). This trial is registered with clinicaltrials.gov NCT02110537.

## 1. Introduction 

Atrial fibrillation (AF) is the most common form of arrhythmia seen in clinical practice. AF prevalence is 0.4–1% in the general population and increases with age. AF prevalence is 3.8% in people >60 years and 9.0% in those >80 years [1, 2].

The American College of Cardiology (ACC) and American Heart Association (AHA) suggest catheter ablation as standard therapy for patients with symptomatic paroxysmal AF who do not respond to single antiarrhythmic drug (AAD) therapy. However, the effectiveness of catheter ablation is low for patients with persistent AF [2–4]. Therefore, the mainstay therapy for these patients has been long-term AAD, even though persistent AF is poorly controlled by AAD therapy. Electrical cardioversion (EC) is also recommended by the ACC when patients do not respond to AADs [3]. However, the ADD preventive effect on AF recurrence after successful EC is substantially low.

Acupuncture is a nonpharmacological therapeutic intervention widely used in the east. Some experimental studies suggest that the mechanism of acupuncture effects on cardiovascular dysfunction including AF, myocardial ischemia, hypertension, and hypotension is pleiotropic, including pain relief, effects on autonomic nervous system and endothelial function, antithrombotic effects, and decrease of oxygenation stress [5, 6]. Similarly, several clinical studies [7–9] showed that acupuncture seemed to be effective for preventing cardiac arrhythmias, but they were of low methodological quality. A recent, small prospective study [10] reported that patients treated with once-per-week acupuncture for 10 weeks after EC were 2.77 times less likely to experience AF recurrence compared to sham acupuncture or no treatment groups. However, there were issues regarding study design and methodology, including AF detection and follow-up. Thus, there is little evidence to recommend acupuncture as an AF treatment option.

Therefore, this trial evaluates the effect of acupuncture on AF recurrence after EC for persistent AF patients who do not respond to AADs.

## 2. Methods/Design

### 2.1. Patient Population and Study Design

This acupuncture in persistent AF (ACU-AF) study will be a multicenter, prospective, participant and assessor blinded, randomized, sham-controlled clinical trial with 2 parallel arms. Two institutes will take part in this study: Kyung Hee University Medical Center and Kangdong Kyung Hee University Hospital. Eighty patients will be enrolled for 2 years. We will compare the efficacy of active acupuncture and sham acupuncture treatments.

**Figure 1:**
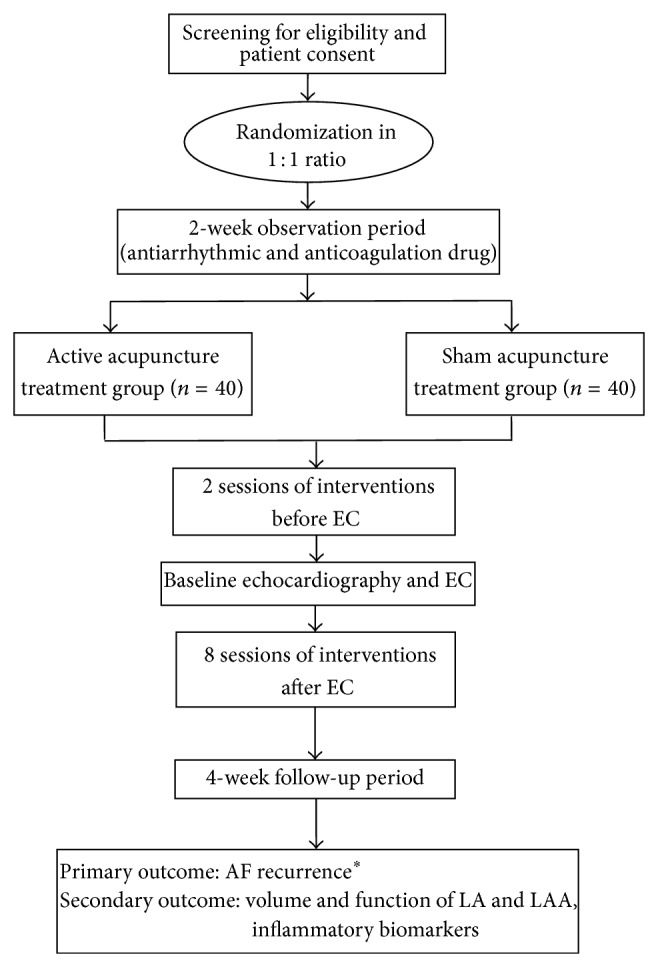
ACU-AF trial flow diagram. Participants receive active acupuncture or sham acupuncture treatment according to the assigned sequence, and AF recurrence is assessed. ^*^AF recurrence is evaluated by 48-hour Holter monitoring, ECG, and f/u echocardiography. AF: atrial fibrillation; EC: electrical cardioversion; ECG: electrocardiography; LA: left atrium; and LAA: left atrial appendage.

Inclusion and exclusion criteria are listed in Table 1. Flow diagram is displayed in Figure 1. The following data will be evaluated at baseline: demographic data, electrocardiographic and echocardiographic data, comorbidities, biomarkers, medications, and CHA2DS2-VASc score. AAD (flecainide 75 mg twice daily) will be administered to patients confirmed with persistent AF lasting more than 7 days. Flecainide administration will be initiated 2 weeks before EC and maintained during the follow-up period. The resistance for flecainide is defined as AF is not converted to sinus rhythm with oral flecainide before EC. Other types of AADs are not allowed, so participants taking other drugs will be required to change their medication to flecainide. Heart rate control drugs will be permitted (calcium channel blockers such as verapamil or diltiazem, except digoxin and beta blocker), and all dose increases or decreases will be determined by an attending physician. Simultaneous anticoagulation therapy with warfarin will be performed to achieve the target international normalized ratio (INR) of prothrombin time (range 2-3) before EC and during the follow-up period. Patients resistant to drug therapy will be randomized into active and sham acupuncture groups through central randomization. After randomization, once-per-week active or sham acupuncture will be initiated 2 weeks before EC.

Transesophageal echocardiography (TEE) will be conducted just before EC to rule out atrial thrombus and evaluate heart function. If any thrombus is present, EC will be delayed for 2 weeks. In this event, TEE will be conducted again after continuous warfarin administration for 2 weeks. If the thrombus disappears in that time, or is not present, EC will be performed.

EC will be conducted with direct-current electrical shocks. Drugs such as propofol or pentothal sodium (1.5 mg/kg) will be used for patient sedation before the procedure [3]. One electrode pad will be located in the 2nd intercostal, to the right of the sternum, and another will be located in the 5th intercostal beside the central axilla line. EC will start at biphasic waveform 100 J and increase to 200 J until restoration of sinus rhythm. If rhythm is converted to sinus rhythm, the patient will receive an additional 8 interventions once per week. If not, the patient will be dropped from the study. But, if clinically acceptable, a second EC will be performed within 2 months after the first.

In summary, this trial will consist of three periods: 2 weeks of observation and medical therapy, 10 weeks of active or sham acupuncture, and 4 weeks of follow-up for a total of 16 weeks. After randomization, participants will receive 10 acupuncture treatments over 10 weeks. Patients will visit the hospital during randomization, every week before EC, 8 consecutive weeks after EC, and 12 weeks after EC. During the follow-up period, patients whose AF is not converted to sinus rhythm with repetitive EC during the follow-up period will be excluded from this study.

### 2.2. Randomization and Blinding

Randomization will be performed using a computer-generated random number list with a permuted block design in a 1 : 1 ratio. Adequate allocation blinding will be ensured by this procedure. After obtaining participant consent, allocation information will be sent to the main investigator at Kyung Hee University Hospital by telephone, and allocations will not be known by the participants or enrolling investigators.

It is almost impossible to blind practitioners due to the nature of the acupuncture intervention; thus they are aware of treatment allocation. Active and sham acupuncture interventions will be conducted as similarly as possible in order to maintain participant blinding. Outcome assessors and statisticians who perform data collection and analysis will all be blinded to participant treatment allocation status. Throughout the study, communications regarding the treatment process will be prohibited.

### 2.3. Intervention

#### 2.3.1. Active Acupuncture Treatment Group

The active acupuncture treatment group will receive two forms of acupuncture: electroacupuncture (EA) and intradermal acupuncture (IDA).

**Figure 2:**
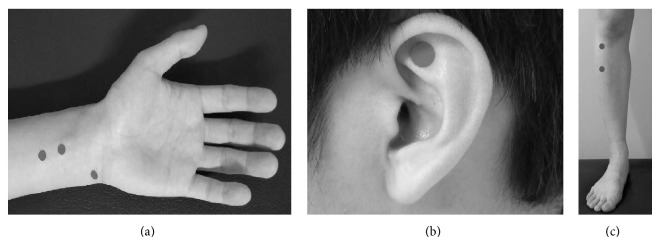
Acupuncture points for active acupuncture group. (a) PC5, PC6, and HT7. (b) TF4. (c) ST36 and ST37.

EA treatment consists of 4 acupuncture points (unilateral PC5, PC6, ST36, and ST37, Figure 2) [11]. Disposable, sterile needles (0.20 mm × 30 mm, Dongbang Acupuncture Inc., Boryung, Korea) and a low frequency electrical stimulator (ES-160, ITO, Japan) will be used. Each needle will be inserted to a depth of 2 ± 0.5 cm at a 90-degree angle. Then, the practitioner will rotate the needle several times and induce de-qi (acupuncture-evoked sensations including numbness, heaviness, soreness, or distention). Thereafter, needles will be connected to the pole, and 2 Hz continuous electric current will be applied for 20 minutes. Intensity will be increased until participants feel stimulation but not discomfort, and their muscles twitch slightly.

Four bilateral acupuncture points (HT7 and TF4, the* shen men* points) have been selected for the IDA treatment (Figure 2) [11]. Disposable, sterile, sticker-type needles (0.18 mm × 1.3 mm × 1.5 mm, Dongbang Acupuncture Inc., Boryung, Korea) will be used. Each needle will be applied to a depth of 1 mm at a 90-degree angle. Attached needles will be replaced every visit by practitioners. However, when the needles accidentally fall out (e.g., when washing their hands or face) or the participants feel some discomfort in the acupuncture sites after returning home, participants will be instructed to replace the needles themselves. They will be fully instructed by the practitioners on how to replace the needles on their first visit. Even if the needles do not fall out and the acupuncture sites are not uncomfortable, practitioners will teach participants to replace the needles every two days to prevent infection. Participants also will be taught that repetitive stimulation of manual pressure is helpful.

Acupuncture treatments will be conducted by Doctors of Korean Medicine educated by the College of Korean Medicine for 6 years who are licensed by the Korean Ministry of Health and Welfare and who have more than 3 years of clinical experience. All practitioners will be trained to follow the study protocol.

#### 2.3.2. Sham Acupuncture Treatment Group

During sham treatment, nonacupuncture points (Table 2) will be superficially stimulated. As with the active treatment, two types of sham acupuncture will be performed: sham EA and sham IDA.

For sham EA, 4 nonacupuncture points (Table 2) located 1.5–2 cm lateral to the real acupuncture points will be punctured to a depth of 0.5 cm at a 90-degree angle. Next, the practitioner will connect needles to the pole, and sounds simulating electroacupuncture will be generated, but electrical stimulation will not be delivered. Manual manipulation will not be performed, nor de-qi sensations stimulated.

For sham IDA treatment, 4 nonacupuncture points have been selected. Locations are listed in Table 2. Each needle will be applied to a depth of 1 mm at a 90-degree angle and participants will also be taught identically with active acupuncture group.

All other elements such as needle size, retaining time, frequency and number of treatments, and practitioner will be identical between the two active and sham acupuncture groups.

### 2.4. Outcome Measurement 

#### 2.4.1. Primary Endpoint

Primary endpoint is AF recurrence rate after EC using regular 12-lead electrocardiography (ECG) and 48-hour Holter monitoring. ECG will be conducted at each visit. Whenever patients feel palpitation, symptoms must be registered and patients must be referred to the hospital. AF recurrence is defined as documented 12-lead ECG or AF episodes exceeding 30 seconds during Holter monitoring. 48-hour Holter will be performed at day 1 after EC at baseline and 1 and 3 months post-EC to evaluate recurrence.

#### 2.4.2. Secondary Endpoints

Secondary endpoints are changes in LA and LAA function and volume using 2- and 3-dimensional echocardiography between baseline and 3 months after EC and changes in inflammatory and endothelial biomarkers (i.e., hs-CRP, NT-pro BNP, interleukin-10 (IL-10), and CD40 ligand) between baseline and 3 months after EC. Factors that make AF recur including demographic data, electrocardiographic and echocardiographic data, comorbidities, biomarkers, medications, and CHA2DS2-VASc score will also be investigated irrespective of active or sham acupuncture treatment.

### 2.5. Sample Size Calculation

According to a prior study [10], AF recurrence rate in the active acupuncture group is expected to be 35.3% and 69.2% in the sham acupuncture group. For 5% significance and 80% power (two-tailed), 33 participants were required for each group. Assuming a dropout rate of 20%, a total of 80 participants with 40 in each group were required.

### 2.6. Statistical Analysis

Demographic data will be analyzed to identify pretreatment equivalencies and differences between the two groups. Continuous variables will be presented as mean ± standard deviation (SD), evaluated for distribution normalcy, and compared using the Student's *t*-test or Mann-Whitney test wherever appropriate. Categorical variables will be presented as frequencies and percentages and compared using chi-squared or Fisher's exact tests wherever appropriate. AF recurrence during the 12-week follow-up will be analyzed with Kaplan-Meier curves.

Multivariate Cox regression analysis will be performed to determine which factor is most strongly associated with AF recurrence. Serious adverse events will be tabulated per treatment group and analyzed using the chi-squared test.

A *P* value less than 0.05 will be considered statistically significant. All analyses will be performed with SPSS software (Windows version 17.0, Chicago, Illinois, USA) by a statistician blinded to participant allocation.

### 2.7. Safety and Adverse Event (AE) Outcomes

Safety and adverse event (AE) outcomes are defined as any unexpected or unfavorable symptoms, signs, or diseases that occur during or after treatment, which are not necessarily caused by the treatment. These AEs include local pain, hematoma, bleeding, redness, itching, dizziness, or local infection. All participants and practitioners will be taught to report any AEs. If there are AEs, the following items will be recorded by practitioners at each visit, and appropriate actions will be taken: type of AE(s), occurrence date, lost date, frequency of occurrence, severity, strength of association with the treatment, acupuncture intervention actions taken, and actions taken with respect to the participant. Serious AEs, such as death or life-threatening events which require urgent intervention, will be reported to the principal investigators immediately, who will determine whether the participants will be dropped from the study.

### 2.8. Ethics

This study protocol was approved by the institutional review boards (IRBs) of Kyung Hee University Hospital (number 1335-04) and is in accordance with the Declaration of Helsinki. Study protocol will be carefully explained in detail to eligible participants, and written informed consent will be obtained from all participants.

## 3. Discussion

This trial was designed to compare active to sham acupuncture efficacy for preventing AF recurrence after EC. Second, to determine which factor is associated with any preventive effects of acupuncture on AF recurrence, changes in LA and LAA volume and function, as assessed by echocardiography and inflammatory biomarkers, will be investigated. We will also use a multivariate Cox regression analysis to investigate factors associated with AF recurrence irrespective of acupuncture or sham intervention.

Previously, Lomuscio et al. [10] performed a randomized, controlled trial of acupuncture for preventing AF recurrence after EC. Patients were randomly assigned to receive acupuncture (ACU group, *n* = 17), sham acupuncture (ACU-sham group, *n* = 13), amiodarone (reference group, *n* = 26), or neither acupuncture nor antiarrhythmic therapy (control group, *n* = 24). Cumulative AF recurrence rates in the amiodarone, ACU, ACU-sham, and control groups were 27%, 35%, 69%, and 54%, respectively (*P* = 0.0075). Therefore, they concluded that acupuncture treatment is as effective as amiodarone for preventing AF recurrence after EC in patients with persistent AF. But this study had some study design and methodology limitations, including AF detection method and follow-up. AF recurrences in this study were determined by spot 12-lead ECG during regular visits or by patient symptom-driven reports. Spot ECG exam underestimates AF recurrence. Also, if the amiodarone and control groups are excluded, acupuncture and sham acupuncture group sample sizes are too small to permit robust statistical generalization. Moreover, delivering no medical therapy to the control group may be an ethical problem and is not applicable in the clinical setting. To overcome these limitations, 48-hour Holter monitoring will be performed in addition to regular ECG exam. All participants will be scheduled to take AADs, and we include only 2 study arms: (1) active or (2) sham acupuncture groups. Additionally, Lomuscio et al. evaluated AF recurrence at 1 year after 10 weeks of acupuncture. However, the long term effect of acupuncture could not be confirmed, since other factors besides acupuncture's effect may have contributed to their results. Even the author of the study could not explain the exact mechanism of acupuncture's long term favorable effect. To address this limitation, we will evaluate clinical outcomes 4 weeks after intervention cessation to decrease the effects of other factors not accounted for in our design.

In the previous study mentioned [10], 3 acupuncture points were used (PC6, HT7, and BL15). In this study, we will include PC6 and HT7 but replace BL15 with ST36, because ST36 has been shown to regulate autonomic nervous system function [12]. Acupuncture points are shown in Figure 2. PC5 and PC6 are located on the pericardium meridian. This meridian is called “guardian of the heart” and is used to treat physical or emotional imbalances, including heart or chest disorders, depression, and anxiety [13]. ST36 and ST37 are the most frequently used stomach meridian points. These are reported to inhibit sympathetic nerve function [12]. Also, recent review on acupuncture's cardiovascular actions presented that EA on PC6, PC7, ST36, and ST37 produced cardiovascular responses [5]. Several experimental studies [14–16] showed that EA at PC5 and/or PC6 may have antiarrhythmic effects compared to sham EA. Other studies suggested that EA at deep somatic nerves, such as the median (PC6) or deep peroneal (ST36) nerves, alleviated sympathetic excitatory-induced heart reactions [17, 18]. According to the textbook of acupuncture [13, 19], HT7 and TF4 have been used to manage various cardiovascular disorders. Many arrhythmia clinical trials [20, 21] also used these acupuncture points. TF4 is one of the most commonly used ear acupuncture points and is located at the triangular fossa apex, a key point in the vagus nerve distribution [22]. This nerve plays an important role in regulating autonomic nervous system function [23, 24]. Acupuncture applied there has been shown to calm the mind and regulate heart function [13, 19].

Also, this study will use EA and IDA together, while previous studies used manual acupuncture (MA) alone [10]. EA was developed from traditional acupuncture and uses electric current. It is more effective than MA for some conditions and offers stronger and more continuous stimulation than MA [25]. IDA is one of many ancient acupuncture needling methods. Needles are left in the intradermal layer for long periods of time and induce continuous stimulation; thus this technique is referred to as “buried acupuncture.” It is generally thought to be effective for chronic diseases which require a consistent stimulus in superficial skin layers [19]. So, we expect to show more powerful and long lasting treatment effects by using EA and IDA together.

## 4. Conclusion

This ACU-AF trial asks if addition of acupuncture to AAD reduces AF recurrence after sinus rhythm conversion by EC in persistent AF patients.

## Figures and Tables

**Table 1:** Inclusion and exclusion criteria of ACU-AF trial.

Inclusion criteria	Exclusion criteria
(1) Persistent AF lasting ≥7 days (2) Aged 20–75 years (3) Resistant with AAD (flecainide) (4) Written informed consent	(1) Age <20 years or >75 years (2) Severe valvular heart disease (3) History of open heart surgery (4) History of treatment for MI within 6 weeks (5) Patients taking or requiring administration of antiviral drugs (6) 2nd degree atrioventricular block or more than two fascicular blocks (7) Severe pulmonary, liver, or renal disease (8) Previous acupuncture treatment for cardiovascular condition within 3 months (9) Patients who are contraindicated to flecainide

AAD: antiarrhythmic drugs; ACU-AF: acupuncture in persistent atrial fibrillation; AF: atrial fibrillation; and MI: myocardial infarction.

**Table 2:** Details of acupuncture points^*^ in active and sham acupuncture treatment.

	Active acupuncture treatment	Sham acupuncture treatment
EA	PC5	Lateral 1.5 cm to PC5 (between PC and LU meridians)
	PC6	Lateral 1.5 cm to PC6 (between PC and LU meridians)
	ST36	Lateral 2 cm to ST36 (between ST and GB meridians)
	ST37	Lateral 2 cm to ST37 (between ST and GB meridians)

IDA	HT7	Lateral to the 2nd extensor digitorum tendon (between TE4 and LI5)
	TF4	Between the jaw point and clavicle point in ear acupuncture

^*^Acupuncture point PC5 refers to the 5th point of pericardium meridian, and ear acupuncture points have different nomenclature (e.g., TF4 means the 4th point in triangular fossa).

EA: electroacupuncture; GB: gall bladder; IDA: intradermal acupuncture; LI: large intestine; LU: lung; PC: pericardium; ST: stomach; and TE: triple energizer.

## Abbreviations

AAD: Antiarrhythmic drug
ACC: The American College of Cardiology
ACU-AF: Acupuncture in persistent atrial fibrillation
AEs: Adverse events
AF: Atrial fibrillation
AHA: American Heart Association
EA: Electroacupuncture
EC: Electrical cardioversion
ECG: Electrocardiography
IDA: Intradermal acupuncture
IRB: Institutional review boards
LA: Left atrium
LAA: Left atrial appendage
MA: Manual acupuncture
SD: Standard deviation
TEE: Transesophageal echocardiography.
